# A cluster-randomised controlled trial to compare the effectiveness of different knowledge-transfer interventions for rural working equid users in Ethiopia

**DOI:** 10.1016/j.prevetmed.2011.02.001

**Published:** 2011-06-15

**Authors:** A.P. Stringer, C.E. Bell, R.M. Christley, F. Gebreab, G. Tefera, K. Reed, A. Trawford, G.L. Pinchbeck

**Affiliations:** a School of Veterinary Science, University of Liverpool, Leahurst Campus, Neston, Cheshire, CH64 7TE, UK; b Royal (Dick) School of Veterinary Studies, University of Edinburgh, Roslin, Midlothian, UK; c Faculty of Veterinary Medicine, Addis Ababa University, Debre Zeyit, P.O. Box 34, Ethiopia; d SPANA, 14 John Street, London, UK; e The Donkey Sanctuary, Sidmouth, Devon, UK

**Keywords:** Randomised controlled trial, Intervention, Education, Equid, Ethiopia, Knowledge transfer

## Abstract

There have been few studies evaluating the efficacy of knowledge-transfer methods for livestock owners in developing countries, and to the authors’ knowledge no published work is available that evaluates the effect of knowledge-transfer interventions on the education of working equid users. A cluster-randomised controlled trial (c-RCT) was used to evaluate and compare the effectiveness of three knowledge-transfer interventions on knowledge-change about equid health amongst rural Ethiopian working equid users. Groups were exposed to either; an audio programme, a village meeting or a diagrammatic handout, all of which addressed identical learning objectives, and were compared to a control group which received no intervention. Thirty-two villages were randomly selected and interventions randomly assigned. All participants in a village received the same intervention. Knowledge levels were assessed by questionnaire administration. Data analysis included comparison of baseline data between intervention groups followed by multilevel linear regression models (allowing for clustering of individuals within village) to evaluate the change in knowledge between the different knowledge-transfer interventions.

A total of 516 randomly selected participants completed the pre-intervention questionnaire, 504 of whom undertook the post-dissemination questionnaire, a follow up response rate of 98%. All interventions significantly improved the overall ‘change in knowledge’ score on the questionnaire compared to the control, with the diagrammatic handout (coefficient (coef) 9.5, S.E. = 0.6) and the village meeting (coef 9.7, S.E. = 0.6) having a significantly greater impact than the audio programme (coef 4.8, S.E. = 0.6). Covariates that were different at baseline, and which were also significant in the final model, were age and pre-intervention score. Although they had a minimal effect on the intervention coefficients there was a significant interaction between age and intervention. This study should aid the design of education materials for adult learning for working equid users and other groups in developing countries.

## 1 Introduction

There are estimated to be 1.8 million horses, 377,000 mules and 4.3 million donkeys working in Ethiopia, the largest population of donkeys in Africa and the second largest donkey population in the world after China (Anon., 2007). Their role in the socio-economics of the country is substantial, with the majority of the Ethiopian population dependent on traditional subsistence agricultural production (DFID, 2006). Livelihoods are predominantly based on agriculture, which accounts for 85% of employment, 45% of national income and over 90% of export earnings, but Ethiopian agriculture still remains low-input, low-value and subsistence-orientated (DFID, 2006). Equids are used for the transportation of goods, people and in some areas for agricultural purposes (Gebreab, 1993; Garuma et al., 2007). They have a direct effect on the lives of rural people by reducing the transport burden of water, fuel wood and goods (Garuma et al., 2007). Although often considered a secondary animal in relation to oxen, donkeys provide an effective entry point for assisting women in both domestic responsibilities and income generating activities (Marshall and Ali, 1997).

Working equids in Ethiopia suffer from low productivity as a result of prevalent parasitic and infectious disease, low nutritional standards and poor management practices (Yilma et al., 1990). These causes contribute to the short life span of Ethiopian donkeys (9–13 years) compared to a possible 37–40 years in the UK (Svendsen, 1994). Due to their perceived low social status, and their single purpose role within farming systems, few farmers keep more donkeys than are needed to fulfil their immediate work requirements (Tesfaye and Smith, 2000). Farmers who do not own a donkey have a relatively weak economic base compared to those who own donkeys, and also own fewer other livestock (Alemayehu et al., 2000).

Wounds are amongst one of the commonest health concerns to afflict working donkeys in many countries (Curran et al., 2005; Pritchard et al., 2005; Biffa and Woldemeskel, 2006; Burn et al., 2007; Sells et al., 2009). In addition, studies of donkeys in Ethiopia have demonstrated that back sores and wounds are the most commonly observed health problem (Tesfaye and Curran, 2005). The majority of these wounds are a result of manmade causes, which is in contrast to the majority of wounds on equids in developed countries that are predominantly due to accidental injury. Wounds in working donkeys are seen on the legs, girth, tail, saddle and wither regions (Pritchard et al., 2005; Sells et al., 2009). These wounds are often caused by a combination of poorly fitting and designed tack or harnesses, beating with sticks and improper management practices (Pearson et al., 2003). Differences in wound severity and location can often be attributed to the different uses of the donkeys: for example, they may be ridden, carry packs or be used for draught, and also to differences in saddle and harness design (Sells et al., 2009).

One approach to decrease the prevalence of wounds is through education of donkey users. Ethiopian farmers have themselves identified a need for greater knowledge through training (Tesfaye et al., 2005). However, there is limited published work available that evaluates the impact of different knowledge-transfer methods on adult learning in developing countries, and to the authors’ knowledge no published work is available that evaluates the effect of knowledge-transfer interventions on the education of working equid users. A variety of knowledge-transfer methods have been widely used for educating people in the developing world including: handouts and leaflets (Tu and Giang, 2002); rural radio (Valente et al., 1994; de Silva and Garforth, 1997; Chapman et al., 2003); drama (Harvey et al., 2000; Soldan, 2004); video (Rimm, 2003); information and communication technology (ICT) (Dawson and Joof, 2005) and community educators (Klepp et al., 1997). However, few studies have utilised randomised controlled trials to assess the impact of knowledge-transfer interventions on their target audience (Eaden et al., 2002; Kelly et al., 2003; Thomas et al., 2003; Bell et al., 2005; Grace et al., 2008). The objectives of this study were to develop a number of different knowledge-transfer interventions for rural working equid users in Ethiopia and, subsequently, to assess the efficacy of these on knowledge change using a c-RCT.

## 2 Materials and methods

### 2.1 Content and design of knowledge-transfer interventions

Both the focus of the education programme, and the design of the knowledge-transfer interventions were informed by a Participatory Situation Analysis (PSA) (Stringer et al., 2009). The PSA gathered information on the perceptions of working equid owners about the health and disease concerns of their animals, and their existing contact and social networks. The PSA identified wounds as an important owner-perceived concern about their donkeys. This information was then triangulated with both clinical records from a veterinary non-governmental organisation (NGO) involved in providing free veterinary care for working equids in Ethiopia, and with available published literature. Results of the triangulation process informed the development of 10 learning objectives (Table 1), which were designed to address key issues associated with wounds and wound management in donkeys, including their causes, sites, treatment, prevention and relevance.

These 10 learning objectives were then incorporated into the design and development of three different knowledge-transfer interventions; an audio programme (A), a village meeting facilitated by an animal health worker (VM) and a diagrammatic handout (HO). The interventions chosen for inclusion in the c-RCT were informed by results of other relevant published studies, and future sustainability, economics and logistical considerations. All interventions were designed with content that was both culturally and socially acceptable, and affordable. Each intervention underwent extensive phases of pretesting, piloting and reverse translation prior to release to study participants. The diagrammatic handout was designed to be predominately image-based with as little text as necessary, having taken into consideration the low levels of literacy and visual literacy identified amongst study participants during the PSA and pretesting phase. The handout consisted of four laminated colour pages of A4 paper (available from author on request), containing high quality photos with background detail removed using Adobe Photoshop CS2. Text on the handout was limited to single words or short sentences in Oromo (regional language of the Oromia region). The handout was distributed to participants on an individual basis at the end of the pre-intervention questionnaire, and was not accompanied by any discussion or clarification of the content.

The audio programme was developed in the format of a short (12 min) radio drama which comprised of a discussion in Oromo between a wise old livestock owner and a young, inexperienced owner, and was recorded by recognised local radio actors. This programme was recorded using digital software (Audacity 1.2.6, http://audacity.sourceforge.net) and was broadcast via an MP3 player and loudspeakers to all participants in a village on a group basis, following administration of the pre-intervention questionnaire. The village meeting consisted of a standardised talk accompanied by both visual ‘poster’ displays and demonstrations given by one local, qualified animal health worker in Oromo. It was delivered to all participants in a village as a group after the pre-intervention questionnaire, and also involved a short question and answer session for clarification at the end of the meeting which could be in either Oromo or Amharic.

### 2.2 Design of c-RCT

A c-RCT design was used to compare the effects of the three knowledge-transfer interventions with a control group (that received no knowledge-transfer intervention) on change in knowledge of equid users. Villages (Kebeles) and rural donkey users were randomly selected and the same intervention was randomly assigned to all participants within each village. Cluster randomisation was necessary to prevent “contamination” between owners belonging to one village via sharing of information. Identical questionnaires were administered both pre- and post-dissemination to assess changes in knowledge levels. Follow up questionnaires were administered 11–18 days post-intervention (median 14, mode 14).

### 2.3 Sample size calculation

Sample size estimates were performed for a clustered design using a cluster sample size calculator (Campbell et al., 2004). An estimate of the variance at village level from previous studies in developing countries (Bell et al., 2005) was used, giving a design effect of 2.3 and an intra-cluster correlation coefficient of 0.14. A total of eight villages each with 15 owners (total 480 participants) per type of intervention were required to detect a 30% change in knowledge (e.g. an increase from a baseline of 20–50%) with 95% confidence and 80% power. Therefore 32 villages with at least 25 owners per village were selected to allow for potential non-response and loss to follow up. A blocked design was used such that, within each set of eight randomly selected villages, each knowledge-transfer intervention and control was assigned randomly to two villages. This was to avoid runs of one type of intervention being selected by chance, as we hypothesized that season, and seasonal job activities of farmers, may affect the response rates.

### 2.4 Study sites and participants

The c-RCT was carried out between November 2008 and July 2009 in one of the seven regional zones of Ethiopia (Oromia). Within this region, one zone (Arsi) was selected based upon: a lack of previous exposure to an equine veterinary NGO, a known high density of donkey users and logistical considerations. Within this area four woredas (administrative departments) (Sire, Hitosa, Tiyo, Degeluna Tijo) were non-randomly selected and a complete list of villages within the woredas was obtained from each woreda agricultural office. Thirty-two villages were randomly selected using random numbers generated in a spreadsheet programme (Microsoft Excel 2007, Microsoft Cooperation, USA). Villages were excluded if there was no road access; the development agent (DA) was deemed too inexperienced or new to that village (were unable to identify and contact randomly recruited participants); if there were inadequate villager records; or if selected villages shared a major market at which contamination may occur. Development agents were recruited to liaise with each selected village and to aid in the participant recruitment process. Lists of village inhabitants were obtained from village agricultural offices or municipality offices, and participants in villages were randomly selected using random numbers generated in Microsoft Excel 2007 (Microsoft Cooperation, USA). Participants were eligible for inclusion in the c-RCT if they were male, owned or used a donkey, over 18 years of age, and able to attend the study visits. All participants were recruited on a volunteer basis and were free to refuse participation or leave the trial at any point. Formal consent was assumed by continued participation in the trial after an introduction to the trial was administered. Once recruited, participants were assigned a unique identification (ID) number and ID card, and were also paid a nominal monetary incentive for participation in each study visit. The visit dates for both the pre-intervention visit and the follow up visit were pre-determined, and DA's were responsible for ensuring that participants were informed of the correct date and time.

### 2.5 Data collection and analysis

Baseline data were collected at the pre-intervention visit and included age, formal education level, radio use, literacy level (Oromo and Amharic), number of donkeys owned, length of donkey use, other animals owned, housing of donkey, exposure to equine veterinary NGO's, position in household, and responsibility for decisions regarding donkey management (use and treatment). Participants’ knowledge-change was measured using 12 concise questions about donkey wounds and wound management. These questions corresponded to the 10 defined learning objectives, and were identical in both pre-intervention and follow up questionnaires. The 12 questions required participants to volunteer between one and four correct responses per question, to achieve a total possible maximum score of 28. Questionnaires were extensively piloted and reverse translated. All questionnaires were administered on an individual basis by one trained animal health worker (AHW) in either of two regional languages (Oromo and Amharic) in a consistent and controlled manner with no additional clarification. Questionnaires took approximately 20 min per participants to complete. To avoid contamination of participants who had not yet received a questionnaire by individuals who had already completed the questionnaire, all participants were kept in two separated groups. This occurred until the time that either a group intervention could be administered (in villages allocated as either audio or village meeting intervention), or participants who had completed the questionnaire could leave the village (in villages allocated as either control groups or handout interventions). The administrators of the questionnaires, and those assessing the outcomes of the trial were not blinded due to logistical constraints involved with this intervention trial.

Data were entered into a spreadsheet (Microsoft Excel 2007, Microsoft Cooperation, USA) and analysed using SPSS v17 (SPSS Inc, Chicago, Illinois, USA) and MLwiN v2.02 (Centre for Multilevel Modelling, Bristol, UK). Data analysis included comparison of baseline-data between intervention groups to check for adequate randomisation using Chi-squared tests for categorical data and Kruskal–Wallis or Mann–Whitney tests for continuous data. The primary outcome measure used was a continuous variable reflecting the change in score between pre-and post-intervention questionnaires. The change in score of individual respondents was compared between the different knowledge-transfer interventions using multilevel linear regression models to allow for clustering of individuals within villages. Analysis was carried out on a per-protocol basis due to no data being available on the outcome of those participants lost at follow up. However due to the small number of participants lost (*n* = 12) this is unlikely to have had a biasing effect. The effect of all covariates that varied at baseline was also considered. Continuous variables (age and pre-intervention score) were centred by subtraction of the sample mean from all observations and checked for linearity before entry into the final model by use of a generalised additive model (GAM) (Hastie and Tibshirani, 1990). A backward-stepwise process was used, with covariates remaining in the model if they were statistically significant (*p* = 0.05), or if they altered the effect of other covariates by greater than 25%. Random coefficients models, allowing the coefficients for fixed effects, including the intervention, to vary across villages (i.e. random slopes), were assessed to determine if the effects varied by villages. The significance of interaction terms was tested between all fixed effect variables. Model diagnostics included detection of villages with a large influence on the fit of the model by examination of residuals and leverage values and evaluation of residuals in normal probability plots to check that the residuals at each level followed a normal distribution (Rasbash et al., 2008).

## 3 Results

### 3.1 Descriptive results

**Fig. 1:**
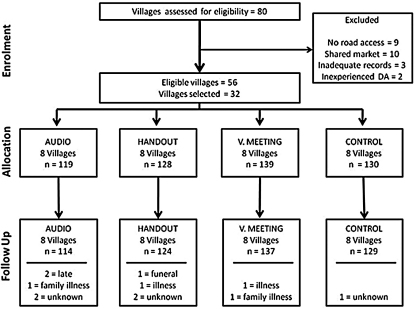
c-RCT flow diagram indicating number of participants and villages at each stage of the trial.

Eighty villages were assessed for eligibility and 24 were excluded due to lack of road access (*n* = 9), the DA was either too inexperienced or new to village (*n* = 2), inadequate villager records (*n* = 3) or due to the sharing of a major market (*n* = 10). From the remaining 56 villages, 32 were randomly selected and interventions randomly assigned (Fig. 1). A total of 516 participants from 32 villages undertook the pre-intervention questionnaire, 504 of these participants undertook the post-intervention questionnaire, a response rate of 98%. Reasons for seven of the 12 participants who were lost at follow up were obtained; these included personal and family illness, funeral attendance and late arrival at the follow up visit (Fig. 1). There was no significant difference in the proportion of participants lost across intervention groups.

Baseline information on participants (Tables 2 and 3) revealed low formal levels of education, with the majority of participants having only attended formal schooling until primary level. The majority owned one donkey, with a large majority owning cattle and oxen as well. Baseline information revealed low levels of literacy, with a greater proportion of participants unable to read Oromo than Amharic. The majority of the participants listened to the radio daily.

### 3.2 Baseline comparison of randomisation

Analysis of baseline data to check the randomisation process showed that a number of variables (age, number of donkeys owned and ownership of horses, sheep, goats and dogs) were significantly different between intervention groups (Tables 2 and 3), and pre-intervention scores approached significance. Further analysis revealed that the pre-intervention scores of the audio group were significantly higher than those of both the handout and village meeting groups. Baseline comparison of age amongst the intervention groups revealed significant differences, with the audio group being older than the control and handout intervention groups. The village meeting intervention group was significantly older than the handout intervention group. The effects of these differences were explored in the multivariable analysis.

### 3.3 Multilevel linear regression analysis

**Fig. 2:**
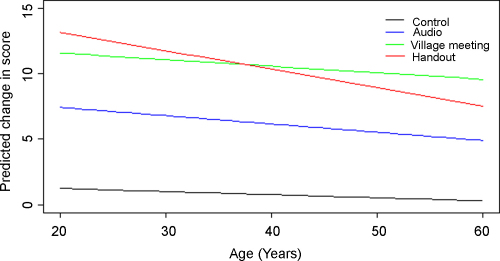
Plot showing the effect of the significant interaction between age and interventions.

**Fig. 3:**
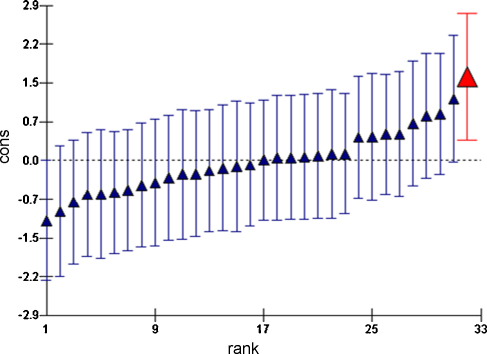
Plot of village level residuals (± 95% confidence intervals) of the 32 villages ordered by rank. The village with the highest residual is shown in bold as a village that received the handout intervention.

Change in score was approximately normally distributed and ranged from −4.5 (i.e. some participants did worse at follow up) to 20. The initial model only considered the interventions (Model 1). All interventions significantly improved the overall change in score between pre- and post-intervention questionnaires compared to the control (Table 4), with the handout and village meeting having a significantly greater impact than the audio programme (*p* < 0.001). There was no significant difference between the village meeting and handout (*p* = 0.4). The final model (Model 2) considered all intervention types and those covariates which were significantly different at baseline comparison. Of these, the only covariates shown to have a significant effect on the outcome were age and pre-intervention score. These both had a linear relationship with the outcome. The higher the pre-intervention score and the older the age of participant, the less the changes in score at follow up. However, there was a significant interaction between age and the effect of the intervention (Fig. 2) showing that the effect of age was more pronounced in the group that received that handout. The variance at the village level was small compared to variance at the individual participant level and accounted for only 4.2% of the total variation, suggesting that the majority of the variance can be attributed to differences between individuals rather than between villages. Random slope effects were not significant, suggesting that there were no differences in the effect of a single type of intervention across different villages. Normal probability plots of both the individual and village level residuals showed that the assumption of normality was reasonable. Village level residual plots (Fig. 3) showed that one village was significantly different from the overall mean. This village received a handout intervention. Plots of leverage and influence values (not shown) also showed that this village had moderately high leverage and the highest influence value. Inclusion of this village as a dummy variable (Rasbash et al., 2008) to fit an intercept separately from those of the other villages did reduce the overall deviance and the parameter estimate for this village was significant (coefficient 3.5, S.E. = 1.1) showing that the change in score was increased in this village. However, estimates for the overall effect of the interventions changed very little.

## 4 Discussion

To the authors’ knowledge this is the first study to evaluate the effectiveness of different knowledge-transfer interventions for adult learning amongst working equid users in a developing country. All interventions improved post-intervention knowledge of the target audience, however, the handout and village meeting improved these scores nearly twice as much as the audio programme. The final model showed that the age of the participant and pre-intervention score (baseline knowledge) had an effect on the outcome variable (change in score). Further, the effect of age varied across interventions and was more pronounced in the participants that received the handout such that the handout performed best amongst young participants, whilst in the older participants the village meeting performed best. This is consistent with previous work, suggesting that older age groups have lower literacy proficiency than younger adults (Desjardins, 2003), most likely as a result of younger populations having received more extended formal schooling than older populations, with the added benefit of having received that schooling more recently. This lower literacy proficiency could lead to a reduced ability for knowledge acquisition from materials requiring literacy and/or visual literacy such as the handout used in this study.

There are few comparable studies in the literature which utilise randomised controlled trials to show the efficacy of different knowledge-transfer interventions, however, our results are supported by evidence of an increase in knowledge with a variety of interventions amongst Tanzanian smallholder dairy farmers (Bell et al., 2005) and of farmer's knowledge of trypanosomosis (diagnosis and treatment) two weeks post-dissemination of an information pamphlet (Grace et al., 2008).

After allowing for the effect of intervention, age and pre-intervention score there was little remaining variation between villages. However, one village was significantly different from the overall mean and this also had a high influence value. Removal of this village had little impact on the estimates of the effects of the interventions (or of age or pre-intervention score). A potential explanation as to why this village had the largest increase in change in score on the questionnaire could be that many participants from this village brought their handouts to the follow up visit (identified from contemporaneous field notes; data not presented), and were therefore able to refer to them prior to (although not during) the questionnaire. It is an advantage of this form of intervention that the content is still available after the initial visit and therefore available to be discussed between villagers at will. However, we did not collect data at this stage to confirm this and the authors are unaware of any specific reasons why this village would be different to other villages in the trial.

The use of defined learning objectives to guide the design and development of all three interventions was essential to ensure that the core content of each was consistent, and that their effect on knowledge-change could then be objectively evaluated (Ramhani, 2006). Learning objectives can provide an explicit overview of both what ‘the learner should have achieved’ and ‘what should be assessed’ at the end of an educational programme, and it is recommended that they are formulated at the start of an educational programme to aid robust curriculum design and delivery (Harden, 2002).

Both the formal education levels and literacy levels of the target audience were expected to be low, and this was considered during the design and development phase of the interventions prior to commencement of the c-RCT. Research carried out during both the PSA and the piloting of interventions identified low levels of literacy ability amongst the target audience in both main regional languages (Oromo or Amharic), suggesting that a more diagrammatic/pictorial intervention was required. The participants in our study revealed low levels of literacy, with a greater percentage of participants unable to read Oromo than Amharic. Despite this, our interventions were designed in Oromo as this is the official language of the region, the language currently being taught at school and the language that the majority of our target audience communicate in (even if illiterate in this language).

There were also concerns over the visual literacy ability of our target audience. Visual literacy relates to the ability of an individual to accurately understand and interpret an image presented to them (Linney, 1995). Images should depict local situations from the daily lives of the intended audience (Harford and Baird, 1997) and should be piloted to ensure that the most suitable image type is utilised (e.g. black and white, colour, background removed, cartoon style). The use of visual images is not the panacea for all low literacy situations as visual illiteracy may be as common as actual illiteracy in some cases (Linney, 1995; Harford and Baird, 1997). Of all of the visual images tested during piloting of interventions, owners were best able to recognise “real-life” photos with the backgrounds removed and whitened out. The success of the handout in this study suggests that the considerable efforts made during the piloting and development phase of the study resulted in the production of a handout that was understandable and appropriate to the visual literacy of the study participants however this did appear to vary with age of the participant. When designing extension materials as knowledge-transfer interventions, consideration must be given to the requirements of the intended target audience. It is important to understand the literacy, visual literacy and formal education levels of the intended target demographic (Bell et al., 2005). Other factors that may need to be considered are age, gender, relevance of content and previous exposure to knowledge-transfer programmes. All of these factors may impact on the decision making process for the design and development of suitable interventions for maximising knowledge change.

In this study, the village meeting and the handout provided the largest change in knowledge of the three study interventions. The combination of an oral presentation with demonstrations and visual images was likely to have accommodated all levels of literacy, visual literacy and language issues. The opportunity for a question and answer session within this format allowed participants to clarify any areas of confusion or any missed messages in either language. The handout was more successful in younger participants than older participants despite being designed to be predominately image-based with as little text as necessary. Older participants may have had lower levels of literacy and visual literacy proficiency, as well as less recent schooling, leading to less knowledge acquisition.

The audio programme was designed to simulate a possible future radio broadcast. Previous studies have shown high radio ownership amongst households within Ethiopia with regular radio listeners (Farr et al., 2005), and this was consistent with our findings which showed that 80% of participants listened to a radio on a daily basis. Formats using a drama performed by local actors were shown to be most popular amongst farmers listening to agricultural extension programmes (Chapman et al., 2003), and the effect of this format was shown to be greatest amongst uneducated (no formal education) individuals (Valente et al., 1994). Although the audio programme in this study had the least impact on change in knowledge when compared to the two other interventions it still significantly improved knowledge. The potential benefits of a successful audio intervention may be the ability to ‘reach’ thousands of listeners with relative ease of administration and low cost, which may outweigh or complement the greater knowledge impacts of the more labour intensive interventions. The strength of rural radio as an extension tool lies in its ability to reach an illiterate audience in a language they understand. Programme format should not simply involve dissemination of technical information, but should understand that the way the target audience discuss problems in their own communities, and provide relevant information in suitable context (Chapman et al., 2003). In this study, despite some participants being unable to volunteer answers to some questions in the post-intervention questionnaire, many owners were able to remember the format and the names of the characters in the audio programme. One owner commented that for this intervention to be ‘successful’ for old men the audio programme should be repeated a number of times.

Only males were selected for inclusion in our study based on pilot work that showed that because of an existing male dominant hierarchy within Ethiopian households, males make the majority of decisions regarding use and healthcare of owned donkeys, and make the majority of decisions regarding household finances. During the piloting and design stage of the study, females were included in both individual and focus group discussions; however, they voiced concern that any new ideas concerning donkeys received from an education programme may not be accepted by the male head of the household (usually their husband). Despite the limitation of only including males in this study, it has been shown that education of the household head (predominately male) is found to decrease risk aversion in the adoption of new innovations in agriculture, thereby increasing the likelihood that a change in behaviour may result after a new and novel education intervention (Knight et al., 2003).

All data in this study were gathered via questionnaire interviews with participants in either of two regional languages (Oromo or Amharic). Although the questionnaires utilised concise questions, many requiring only single word answers, the accuracy of all the data must be considered carefully, especially as the information required by the authors was translated. Reliability of participant information was not validated and may be imperfect or biased by participant reporting of perceived correct answers. However due to the study design (c-RCT), we would expect this bias or measurement error to be randomised across all participants, in all intervention groups, and therefore to have minimal effect on the estimates of the effects of interventions. Randomisation is designed to equally distribute potentially confounding factors across intervention groups; whether or not to present adjusted or unadjusted results is a subject of active debate (Dohoo et al., 2003). The authors are unaware of any reason why the randomisation process in this study was not completely successful, and whether increasing the number of villages would have achieved complete randomisation. Results presented in this study have been adjusted for potential confounders and are presented in Model 2 which shows that they had a minimal effect on the interventions. Use of a control group allowed us to monitor participants for the ‘Hawthorne Effect’ (that a participant in the control group receiving a pre-intervention questionnaire would subsequently have a greater level of knowledge at the follow up visit) (Campbell et al., 1995). Little evidence of the ‘Hawthorne Effect’ was seen in this study as the average improvement in the control group was only 0.6 marks.

The three distinctly different knowledge-transfer interventions were utilised in this trial with varying levels of success. This highlights the need for tailor-made interventions specifically designed for the intended target audience to maximise knowledge change. It is worth noting that this study only intended to measure a change in knowledge of the participants. No attempt was made to measure whether this change in knowledge subsequently led to a change in behaviour. There are many reasons that interventions may fail to lead to a change in knowledge (such as poorly designed interventions, or literacy issues) as described earlier, however knowledge-transfer interventions may also fail to result in behavioural change for a number of reasons: limited access to the knowledge, the information (as presented) may be at odds with currently held beliefs; the presented material may be poorly understood; there may be financial constraints to implementation; there may be containment of ideas within groups, or inappropriate presentation of the information and therefore poor understanding. Consequently, effective animal health knowledge may remain unused by owners. The knowledge-transfer programme carried out in this study may be considered as an initial step towards behaviour change; with other components such as skills development, attitudes development and motivational support also being required (Mitchell et al., 2001). Further work to evaluate longer term knowledge retention and owner-reported behaviour change is now underway.

## 5 Conclusion

In future, knowledge-transfer interventions developed for rural equid users in this region of Ethiopia should consider the formal education level, literacy/visual literacy ability and age of audience as key issues, and should be thoroughly piloted and refined before final release. This study showed that direct contact with a specifically trained animal health worker, in combination with a mixture of demonstration, presentation and question and answer session was the most effective knowledge-transfer method. However, interventions based on visual images, designed and piloted with the intended audience were also shown to be successful, particularly in younger age participants. Ethiopia, with its large population of working equids, is ideally placed to benefit from appropriate education or extension programmes for the owners and users of equids. The results from this study may be beneficial to other populations of livestock owners, particularly in sub-Saharan Africa, however, it is likely that different issues associated with learning across different communities may exist, and these must be carefully considered when designing education programmes.

## Conflict of interest statement

None declared.

## Figures and Tables

**Table 1:** Learning objectives used to develop knowledge-transfer interventions on the topic of wounds and wound management in donkeys in Ethiopia.

	Learning objectives
1	Be able to list 4 causes of manmade wounds.
2	Identify 4 common sites/areas affected by manmade wounds.
3	Be aware of good and bad topical treatments for wounds.
4	Describe how to prepare an appropriate salt solution for cleaning wounds.
5	Be able to list 3 steps involved in cleaning wounds appropriately.
6	Recognise 2 signs of an early harness wound.
7	Select appropriate material as a base layer for the harness.
8	Describe 3 important features of the padding on the harness.
9	Describe an important feature of harness base layer care.
10	Recognise 3 disadvantages of your donkey having wounds.

**Table 2:** Age and pre-intervention score, and comparison across intervention groups for 516 participants in a c–RCT in Oromia region, Ethiopia.

Variable		Overall	Intervention				Kruskal–Wallis *p* value
			Control	Audio	Handout	Village meeting	
Age	Mean	45.66	44.45	48.85	42.27	47.01	0.01
	Median	45.00	43.00	48.00	40.00	48.00	
	Percentiles (25)	33.00	33.00	36.00	32.00	32.00	
	Percentiles (75)	57.00	54.25	60.00	50.75	60.00	

Pre-intervention score	Mean	6.28	6.40	6.69	5.95	6.10	0.08
	Median	6.00	6.75	7.00	6.00	6.00	
	Percentiles (25)	5.00	5.00	5.00	5.00	4.00	
	Percentiles (75)	8.00	8.00	8.00	7.00	8.00	

**Table 3:** Baseline information and comparison across intervention groups for categorical data for 516 participants in a c–RCT in Oromia region, Ethiopia.

Variable		Overall (%)	Intervention				Chi square *p* value
			Control *n* (%)	Audio *n* (%)	Handout *n* (%)	Village meeting *n* (%)	
Education level	No education	24.6	26 (20)	30 (25)	34 (27)	37 (27)	0.6
	Adult education only	14.5	14 (11)	21 (18)	19 (15)	21 (15)	
	Primary	33.3	49 (38)	33 (28)	40 (31)	50 (36)	
	Junior	13.8	19 (15)	18 (15)	15 (12)	19 (14)	
	Higher	13.6	22 (17)	17 (14)	19 (15)	12 (9)	
	Other (advanced)	0.2	0 (0)	0 (0)	1 (1)	0 (0)	

Literacy (Oromo)	No	78.5	101 (78)	99 (83)	102 (80)	103 (74)	0.4
	Yes	21.5	29 (22)	20 (17)	26 (20)	36 (26)	

Literacy (Amharic)	No	44.6	50 (39)	53 (45)	56 (44)	71 (51)	0.2
	Yes	55.4	80 (62)	66 (56)	72 (56)	68 (49)	

Listen to radio daily	No	20.0	25 (19.2)	23 (19.3)	19 (14.8)	36 (25.9)	0.2
	Yes	80.0	105 (80.8)	96 (80.7)	109 (85.2)	104 (74.1)	

Number of donkeys	0	6.0	6 (4.6)	8 (6.7)	7 (5.5)	10 (7.2)	0.04
	1	52.1	60 (46.2)	79 (66.4)	60 (46.9)	70 (50.4)	
	2	27.9	43 (33.1)	22 (18.5)	41 (32.0)	38 (27.3)	
	3	10.3	19 (14.6)	6 (5.0)	13 (10.2)	15 (10.8)	
	>3	3.7	2 (1.5)	4 (3.4)	7 (5.5)	6 (4.3)	

Own horse	No	71.1	71 (54.6)	76 (63.9)	109 (85.2)	111 (79.9)	<0.001
	Yes	28.9	59 (45.4)	43 (36.1)	19 (14.8)	28 (20.1)	

Own mule	No	97.5	124 (95.4)	116 (97.5)	128 (100)	135 (97.1)	0.1
	Yes	2.5	6 (4.6)	3 (2.5)	0 (0)	4 (2.9)	

Own cattle/ox	No	6.4	5 (3.8)	6 (5.0)	11 (8.6)	11 (7.9)	0.3
	Yes	93.6	125 (96.2)	113 (95.0)	117 (91.4)	128 (92.1)	

Own sheep	No	37.0	33 (25.4)	37 (31.1)	69 (53.9)	52 (37.4)	<0.001
	Yes	63.0	97 (74.6)	82 (68.9)	59 (46.1)	87 (62.2)	

Own goat	No	74.2	107 (83.3)	96 (80.7)	82 (64.1)	98 (70.5)	<0.001
	Yes	25.8	23 (17.7)	23 (19.3)	46 (35.9)	41 (29.5)	

Own dog	No	27.9	18 (13.8)	35 (29.4)	36 (28.1)	55 (39.6)	<0.001
	Yes	72.1	112 (86.2)	84 (70.6)	92 (71.9)	84 (60.4)	

Own poultry	No	21.9	30 (23.1)	25 (21.0)	22 (17.2)	36 (25.9)	0.4
	Yes	78.1	100 (76.9)	94 (79.0)	106 (82.8)	103 (74.1)	

**Table 4:** Multilevel linear regression models showing the impact of different interventions on a change in score between questionnaires in 504 participants in a c-RCT in Oromia region, Ethiopia.

	Model 1		Model 2	
	Coefficient (S.E.)	*p*-value	Coefficient (S.E.)	*p* value
Intervention				
Control (intercept)	0.6		0.6	
Audio	4.8 (0.6)	<0.001	5.2 (0.6)	<0.001
Handout	9.5 (0.6)	<0.001	8.9 (0.6)	<0.001
Village meeting	9.7 (0.6)	<0.001	9.6 (0.5)	<0.001
Age (years)^a^			−0.02 (0.02)	0.2
Pre-intervention score^a^			−0.4 (0.03)	<0.001
Intervention × Age				
Control × Age			Ref.	
Audio × Age^a^			−0.04 (0.03)	0.135
Handout × Age^a^			−0.12 (0.03)	<0.001
Village meeting × Age^a^			−0.03 (0.03)	0.3
Village variance	0.5 (0.3)		0.7 (0.3)	
Individual variance	10.7 (0.7)		8.8 (0.6)	

Ref.: reference category.

a Indicates variables were centred. Therefore the control coefficient (intercept) represents the change in score for controls of average age and with average pre-intervention score.
